# Metabotyping the Welsh population of badgers based on thoracic fluid

**DOI:** 10.1007/s11306-022-01888-6

**Published:** 2022-05-07

**Authors:** James Scott-Baumann, Richard Pizzey, Manfred Beckmann, Bernardo Villarreal-Ramos, Jonathan King, Beverley Hopkins, David Rooke, Glyn Hewinson, Luis A. J. Mur

**Affiliations:** 1 Institute of Biological, Environmental and Rural Science, Aberystwyth University, Aberystwyth, SY23 3DA Ceredigion UK; 2 Centre of Excellence for Bovine Tuberculosis, Aberystwyth University, Ceredigion, SY23 3AR UK; 3 Wales Veterinary Science Centre, Y Buarth, Aberystwyth, SY23 1ND Ceredigion UK; 4 ProTEM Services Ltd, West Sussex, UK; 5 TB Research Group, Animal and Plant Health Agency, New Haw, Addlestone, KT15 3NB Surrey UK; 6 B2.03 Edward Llwyd, Penglais, Aberystwyth, SY23 3FL UK

**Keywords:** Badgers, High resolution metabolomics, Metabotyping, Diagnostics, All wales badger found dead

## Abstract

**Introduction:**

The European badger (*Meles meles*) is a known wildlife reservoir for bovine tuberculosis (bTB) and a better understanding of the epidemiology of bTB in this wildlife species is required for disease control in both wild and farmed animals. Flow infusion electrospray—high-resolution mass spectrometry (FIE-HRMS) may potentially identify novel metabolite biomarkers based on which new, rapid, and sensitive point of care tests for bTB infection could be developed.

**Objectives:**

In this foundational study, we engaged on assessing the baseline metabolomic variation in the non-bTB infected badger population (“metabotyping”) across Wales.

**Methods:**

FIE-HRMS was applied on thoracic fluid samples obtained by post-mortem of bTB negative badgers (n = 285) which were part of the Welsh Government ‘All Wales Badger Found Dead’ study.

**Results:**

Using principal component analysis and partial least squares—discriminant analyses, the major sources of variation were linked to sex, and to a much lesser extent age, as indicated by tooth wear. Within the female population, variation was seen between lactating and non-lactating individuals. No significant variation linked to the presence of bite wounds, obvious lymphatic lesions or geographical region of origin was observed.

**Conclusion:**

Future metabolomic work when making comparisons between bTB infected and non-infected badger samples will only need be sex-matched and could focus on males only, to avoid lactation bias.

**Supplementary Information:**

The online version contains supplementary material available at 10.1007/s11306-022-01888-6.

## Introduction

Bovine tuberculosis (bTB), a chronic bacterial disease caused by *Mycobacterium bovis*, poses the most significant infectious disease threat to cattle farming in the UK. The control of bTB has significant financial costs in the UK of roughly £150 million per year to government and industry (Defra, 2020). Control measures include: regular herd testing (skin tests) which result in slaughter of infected animals and increased controls on movement of cattle, as well as surveillance of carcasses at slaughter for lesions. In recent years however the reduction of bTB in the UK has slowed, despite these control measures. Potential complicating factors against complete eradication of the disease include: the limited sensitivity of the current skin tests, biosecurity on farms, movement of cattle through trade, as well as wildlife reservoirs (Godfray et al*.*, 2018).

Many mammalian species, both domestic and wild, can become infected and act as reservoirs for *M. bovis* (Broughan et al., 2013). In the UK these include: boar, deer, foxes and badgers (Delahay et al., 2007). *M. bovis* infection was first discovered in the European badger *Meles meles* in Britain in 1971, and it has since been shown to be endemic and widespread in badgers across the British Isles (Jenkins et al., 2008; Murphy et al., 2010; Schroeder et al., 2020). They have been a protected species since 1992; interference them or their setts is illegal (*The Protection of Badgers Act*, 1992). The high prevalence of badgers on farming land and their omnivorous scavenging habits can lead to close contact with cattle (Garnett et al., 2002). This behaviour of badgers scavenging for food on farms, alongside the long survival time of *M. bovis* in the environment could facilitate both direct and indirect transmission routes between cattle and badgers (Ghodbane et al., 2014; Godfray et al*.*, 2018; Krebs et al., 1997; Young et al., 2005).

Multiple studies have utilised road-killed badgers as a way of monitoring bTB in the wild badger population through post-mortem and identification of *M. bovis* (Abernethy et al., 2011; Goodchild et al., 2012; Sandoval Barron et al., 2018; Schroeder et al., 2020). The co-localisation of *M. bovis* spoligotypes (Goodchild et al., 2012) and genotypes (Schroeder et al., 2020) in cattle and badgers was shown based on road-killed badgers in Wales. It was also shown that an infected badger was 12.3 times more likely to be within 5 km of a confirmed cattle bTB breakdown than an uninfected badger (Goodchild et al., 2012). Bacterial whole genome analysis of *M. bovis* has provided additional information on the directionality of this interaction, showing that badger to cattle transmission occurs more frequently than cattle to badger, but that intra-species transmission occurs more frequently for both cattle and badgers than inter-species transmission (Crispell et al., 2019).

Eradicating bTB in badger populations therefore requires accurate tests that could allow targeted management of infected setts or individuals. Current tests available for the diagnosis of bTB on blood samples from live trapped badgers include: PCR-based approaches, the Brock test (indirect ELISA), the BrockTB Stat-Pak assay, IFN-γ immune assays, IgA ELISAs and detection of the P22 multiprotein complex derived from the purified protein derivative (PPD) of bTB by ELISA (Buzdugan et al., 2017; Chambers et al., 2010; Dalley et al., 2019; Infantes-Lorenzo et al., 2019; King et al., 2015). These tests however are limited in use by the time and laboratory equipment required to process them. This has significant cost and animal welfare implications when dealing with a wild, trapped animal, making them impractical. One solution could be a point-of-care (POC) test, such as that used in Chambers et al. (2010) or Stewart et al. (2017), however with sensitivities quoted as 35% and 8% respectively, these may have limited use. Therefore, there is the need for a new rapid and sensitive POC test for bTB in badgers. Metabolomics could hold the key to identifying novel biomarkers that could be used in the development of such a test. Metabolomics is an approach whereby 1000 s of metabolites can be rapidly assessed in a biological sample. The speed and ease of identifying metabolites has increased in recent years through the development of tools such as high-resolution mass spectrometric platforms and bioinformatic analysis pipelines (Segers et al., 2019). As the metabolome integrates genomic and proteomic changes it may more accurately reflect the status of an organism, organ or cell in response to, for example, pathogenic challenge (Wishart, 2019). However, before metabolomic comparisons can be made between bTb infected and non-infected badgers, we must have an understanding of what baseline variation there is in the population. Particularly any significant sources of variation such as age or sex, which may have to be controlled for.

Here, flow infusion electrospray high resolution mass spectrometry (FIE-HRMS) is used to investigate differences in the small metabolite (< 1.5 kDa) constituents of thoracic fluid samples from badgers found dead in Wales that are bTB negative, based on culture of lymph nodes collected at post-mortem. By making comparisons between the different aspects of all available metadata collected at post-mortem (such as age, sex, geographical region, and lactation status) (see Table S1) we can identify some major sources of variation in the badgers’ metabolomes. We observed sex was the major source of variation and, within females, lactation status. These variables need to be controlled for in future work aiming to identify metabolite biomarkers for bTB infection in badgers.

## Materials and methods

### Sample recruitment and collection

The badgers (n = 285) involved in this study were collected dead on the side of the road as part of an ongoing surveillance study by Welsh Government and the Animal Plant Health Agency (APHA), the all wales badger found dead study (AWBFD study). Badgers reported by members of the public were collected and brought to a laboratory where they were deemed appropriate for post-mortem (PM) if they are intact, not distended with gas, with no severe myiasis and were not frozen. Carcasses spend no more than four days in cold storage before PM. PM involves an external examination which includes: sexing, approximate aging based on dental wear, and checking for lactation if female. Aging badgers on tooth wear in this way has been shown to have a 71% accuracy to within 1 year (Delahay et al., 2011). Badgers were scanned for microchips as well as clipping of guard hairs or any colour marker to indicate historical trapping and vaccination. Any signs of external injury, bite wounds, illegal trapping or snaring are also noted. Internal examination focussed on identification of any gross lesions and sampling of tissues for mycobacterial culture. Detailed examination is made of the pericardial sac, lungs, liver and kidneys including internally by making several, longitudinal incisions across each. Each lymph node is incised at least once and a pool of sample material is created, containing retropharyngeal, bronchial lymph nodes, mediastinal and hepatic lymph nodes. Pool two contains a section of any bite wound or any internal visible lesions suggestive of tuberculosis. All badgers designated as bTB positive were done so based solely on the results of culture. For this study, only culture negative badger thoracic fluid samples were used.

Thoracic fluid samples were the liquid obtained from the badger’s thoracic cavity after removal of the pluck (heart, lungs and trachea). The viscosity, colour and transparency of these thoracic samples varied greatly (Fig. S1; supplementary material). They are likely to represent a mixture of frank blood, tissue fluid, and any lymph or pleural effusion. Samples were collected into 2 mL tubes and frozen at − 80 °C until further analysis.

### Flow infusion electrospray mass spectrometry (FIE-MS)

All samples were processed for metabolomics in a blinded manner. 200 μL of each sample was added to 1520 μL of 4:1 (v/v) mix of MeOH:chloroform (HPLC grade) and 50 mg of acetone-washed glass beads (< 160 μg, Sigma, UK). These were vortexed, shaken (15 min at 4 °C), left to settle (− 80 °C for 20 min), then centrifuged (1800× g for 10 min). 100 μL of the supernatant was transferred to a glass vial with glass insert, sample injection order was randomised to reduce batch effects and run on an ExactiveTM Orbitrap Mass Spectrometer (Thermo Scientific) as described in Baptista et al. (2018). No internal chemical standards were used in this study but instead calibration throughout the run is performed using master mixes, of each sample combined, and blanks, consisting of the extraction solvent mixture.

### Statistical analysis

Principal component analyses (PCA) and partial least square discriminant analysis (PLS-DA) analyses were performed using the MetaboAnalyst 4.0 platform (Pang et al., 2021). Raw data were log-transformed and Pareto-scaled before analysis, and outputs were Bonferroni-corrected for multiple comparisons. Major sources of variation were identified using *t-*tests or variable importance projection (VIP) scores. Significant *m/z* were tentatively identified using the MZedDb database (Draper et al., 2009) based on predicted masses *m/z* identified and likely ionisation forms. In MetaboAnalyst 4.0 pathway analysis (integrating enrichment analysis and pathway topology analysis) incorporating the mummichog algorithm was used for biochemical pathway enrichment analysis. The raw data are available on Mendeley Data (https://doi.org/10.17632/8m5n462gcv.1). The metabolomics standards initiative (MSI) minimum reporting standards for environmental metabolomics studies has been followed (Table S1).

## Results

**Fig. 1:**
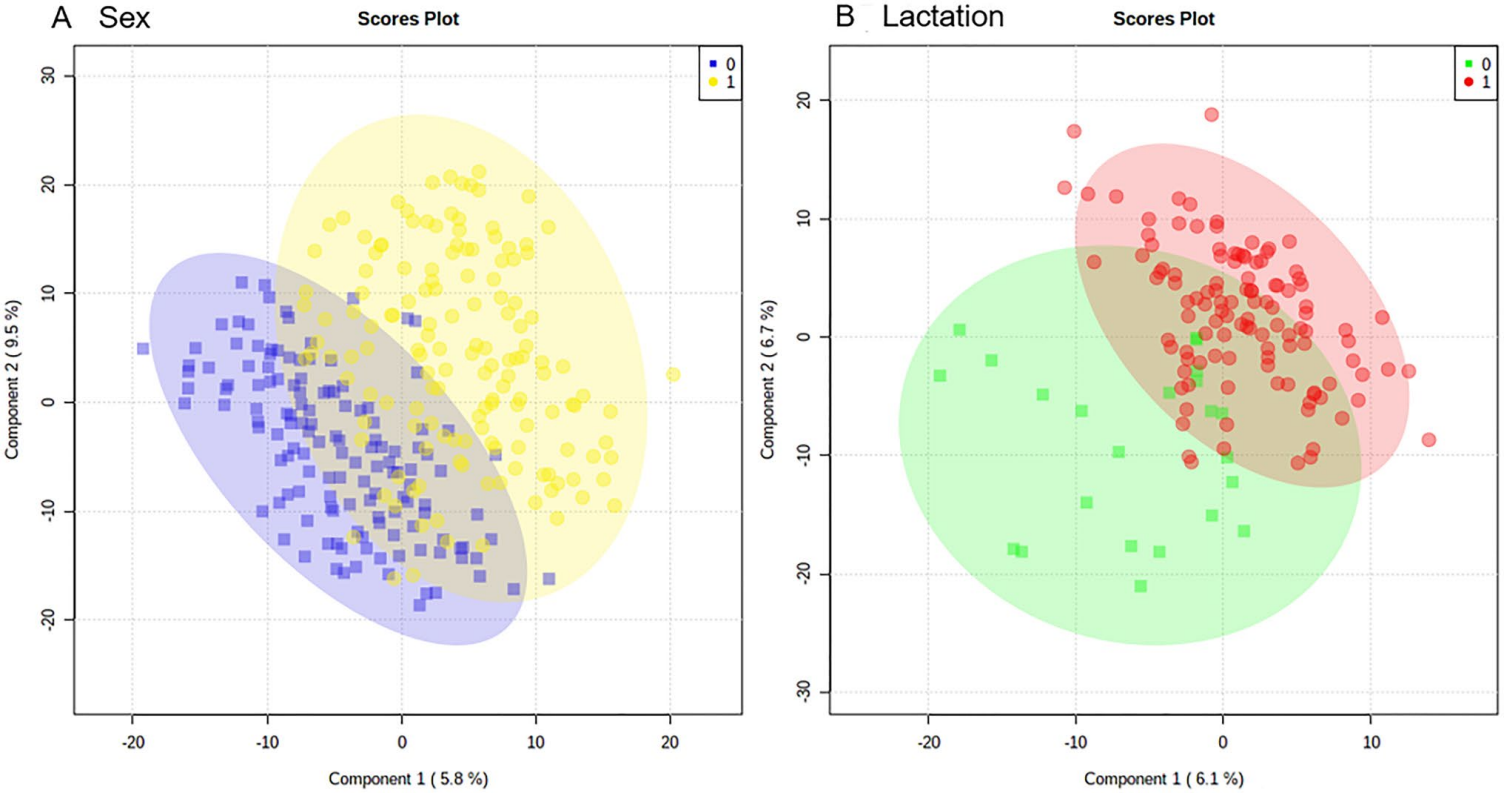
Partial Least Squares Discriminant analysis of thoracic fluid metabolomes comparing badgers based on **A** sex (0 = female, 1 = male) or **B** lactation status, (0 = without, 1 = with). The shaded ellipsis represent 95% CI for each group

**Fig. 2:**
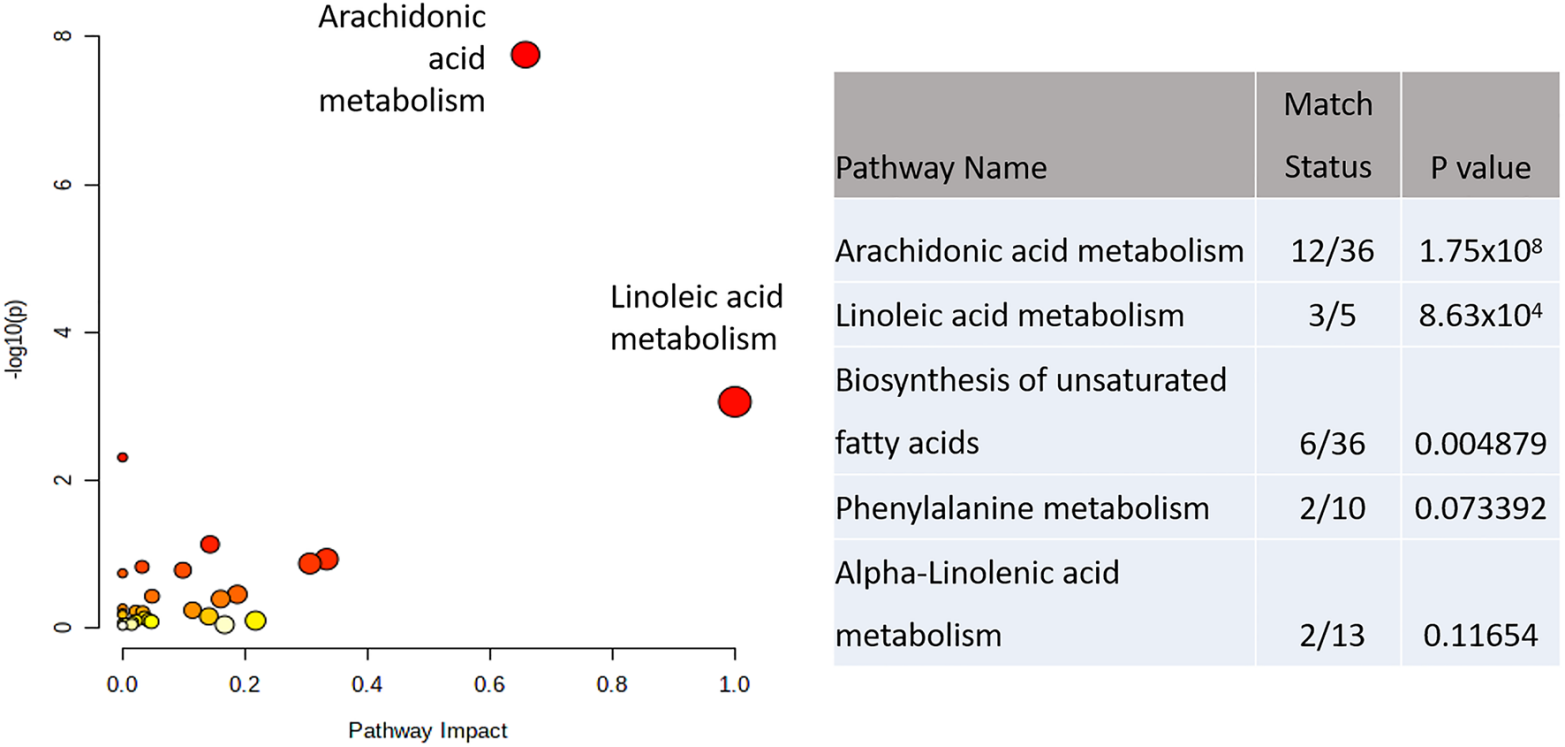
Enhanced pathway analysis indicating metabolomic differences in thoracic fluid metabolomes comparing badgers based on sex

**Fig. 3:**
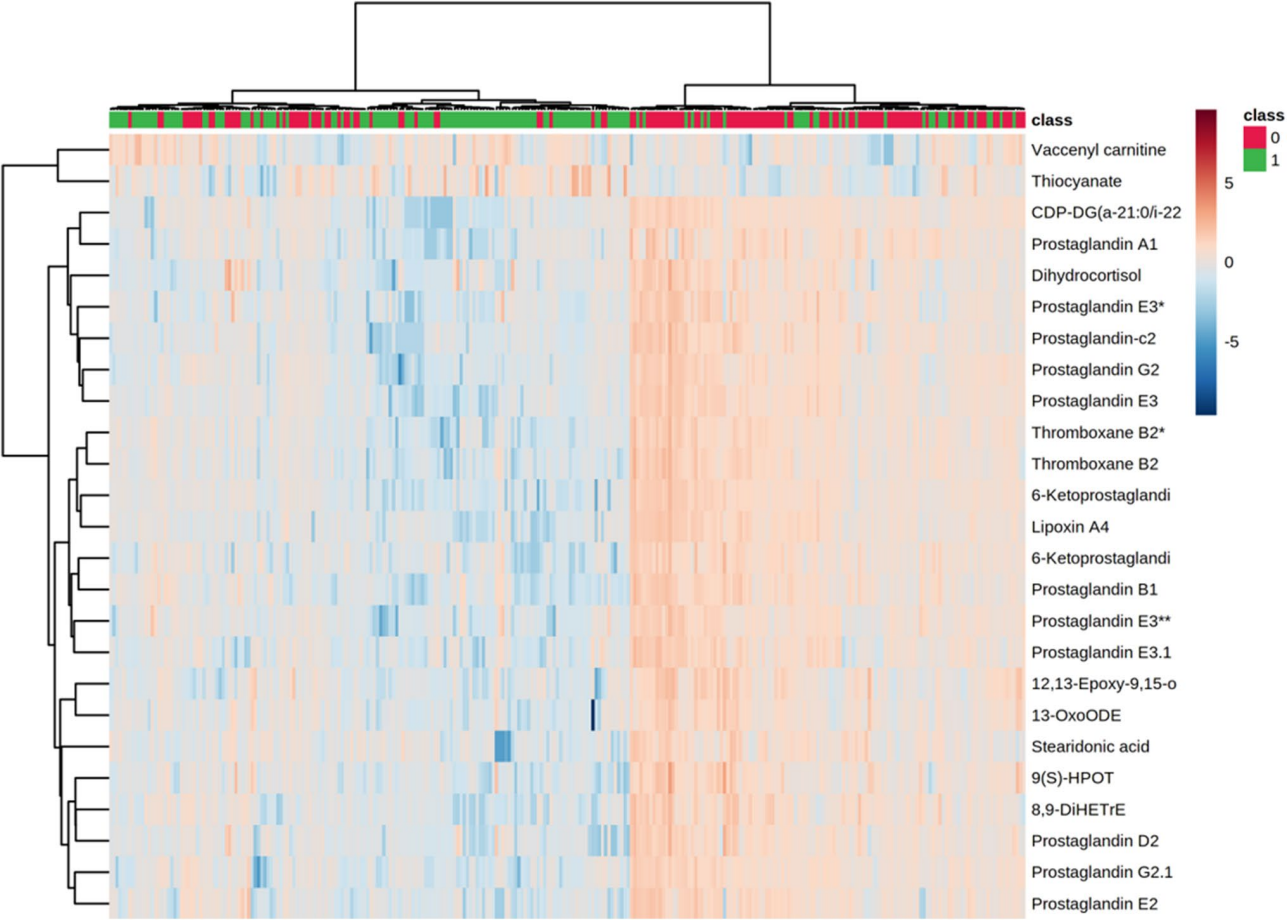
Heatmap showing metabolites which accumulated to different levels in thoracic fluid metabolomes when comparing sex (0 = female, 1 = male)

Initial assessments of the badger metabolomes using PLS-DA indicated that the metabolomes of male and female badgers could be separated (Fig. 1A). Further, when the female population was further stratified into lactating and non-lactating individuals, the metabolomes were shown to be clearly different (Fig. 1B). The sources of variation linked to sex were targeted by *t*-tests and then assessed by the mummichog programme which produced both tentative identifications of the *m/z* and linked these to biological pathways (Xia, 2017). This showed the sex-linked *m/z* variables in thoracic samples were associated with arachidonic acid and linoleic acid metabolism (Fig. 2). The tentatively identified *m/z* were extracted from the data matrix and compared using a heatmap (Fig. 3). This indicated that the identified prostaglandins, thromboxanes, leukotrienes, linoleic acid and arachidonic acid broadly displayed increases in samples from female compared to male carcasses.

**Fig. 4:**
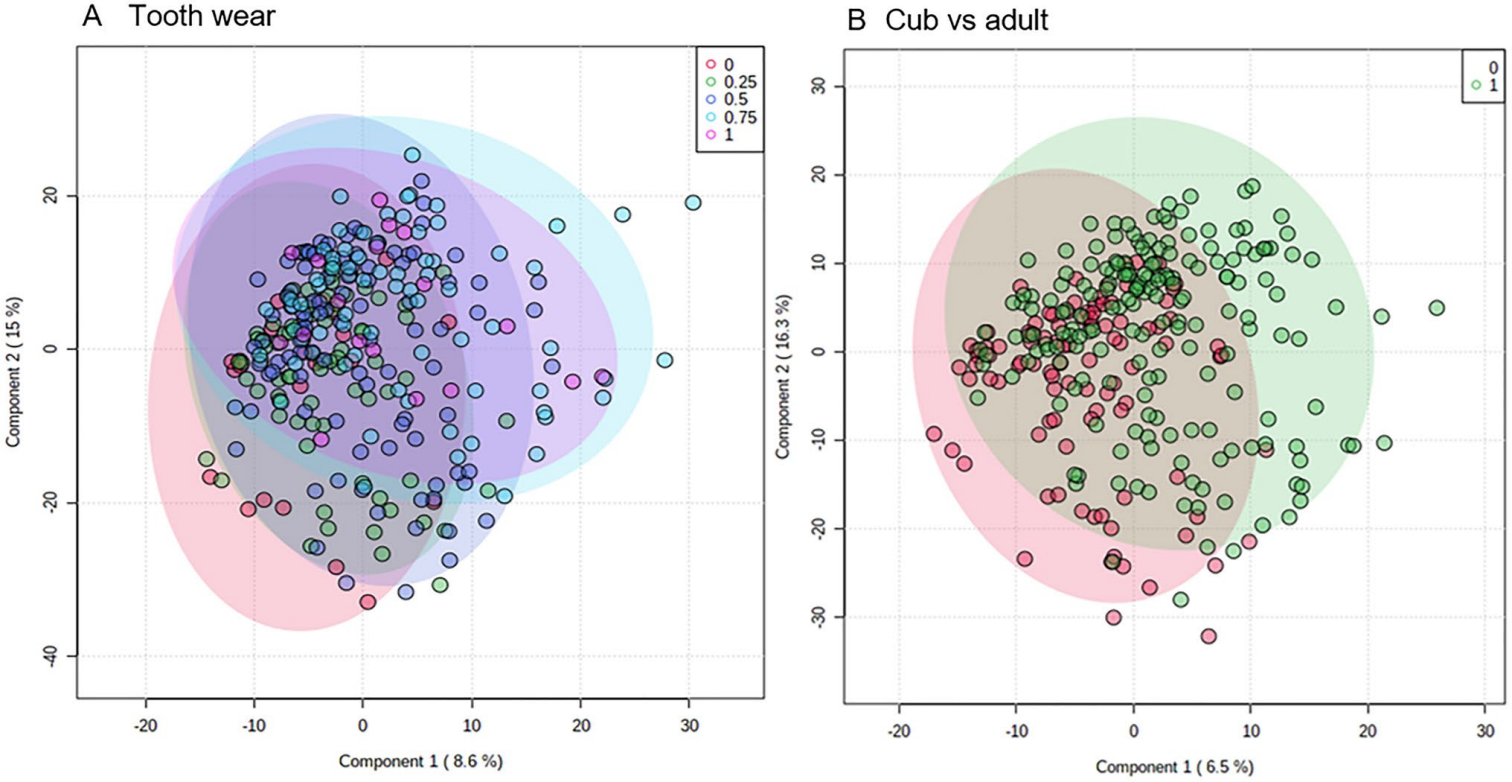
Partial Least Squares Discriminant analysis of thoracic fluid metabolomes comparing badgers based on age. Age as indicated by (**A**) grade of tooth wear (0, 0.25, 0.5, 0.75 and 1) and **B** differentiation into adults and cubs. The shaded ellipsis represent 95% CI for each group

Next, it was considered how badger age could influence the thoracic fluid metabolomes. One way that this can be assessed is by using tooth wear categories 0 and 0.25, which represent cubs, against the other three categories representing adult badgers. Using these five categories, PLS-DA showed very little separation associated with tooth wear (Fig. 4A). However, given that categorisation by tooth wear is subjective, potential metabolomic differences between the broader age categories of cub and adult were investigated (Fig. 4B). PLS-DA suggested some separation between the thoracic fluid of cub and adult metabolomes. A possible over-lap or interaction between sex and age was investigated by two way ANOVA of the 3304 m*/z* features with the significant features displayed using a Venn diagram (Fig. S2). This indicated that most significant (*P* < 0.05) differences were linked to sex (349) as opposed to age (50). Only 2 were shared between sex and age, 5 features were only seen through the interaction with sex and age and 25 shared with all three categories.

**Fig. 5:**
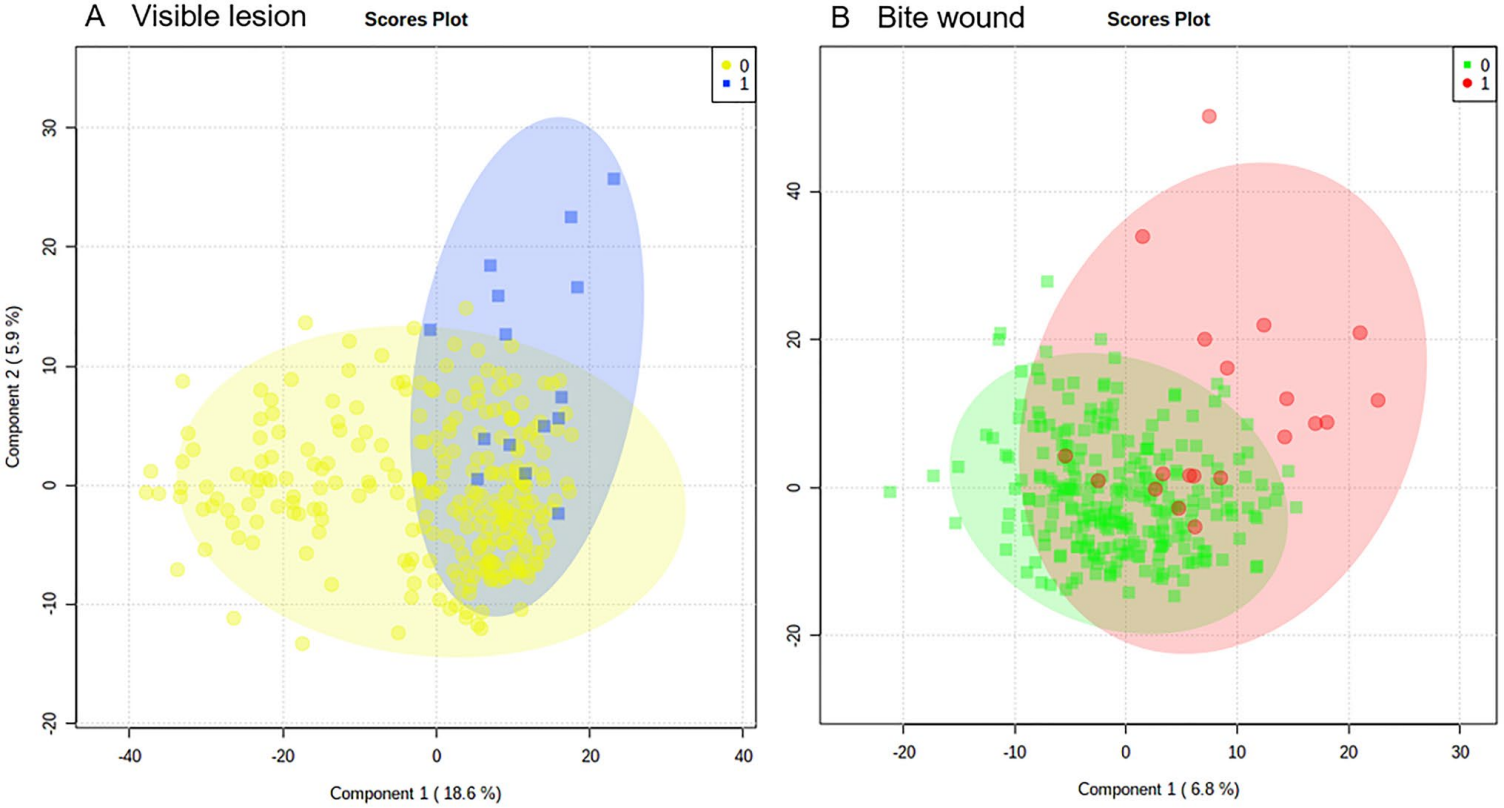
Partial Least Squares Discriminant analysis of thoracic fluid metabolomes comparing badgers with **A** visible lesions (0 = without, 1 = with) or **B** bite wound (0 = without, 1 = with). The shaded ellipsis represent 95% CI for each group

Further assessments of the badger metabolomes focused on the presence of bite wounds or any visible lesions in their lymph nodes as these could be expected to have a significant impact on thoracic fluid samples. In particular, visible lymphatic lesions (which went on to be culture negative for *M. bovis*) could indicate inflammatory or non-bTB infectious conditions occurring resulting in a lymphadenopathy. However, PCA and PLSDA indicated that the metabolomes show little separation between badgers based on the presence of any visible lesions in their lymph nodes (Fig. 5A) or bite wounds (Fig. 5B).

**Fig. 6:**
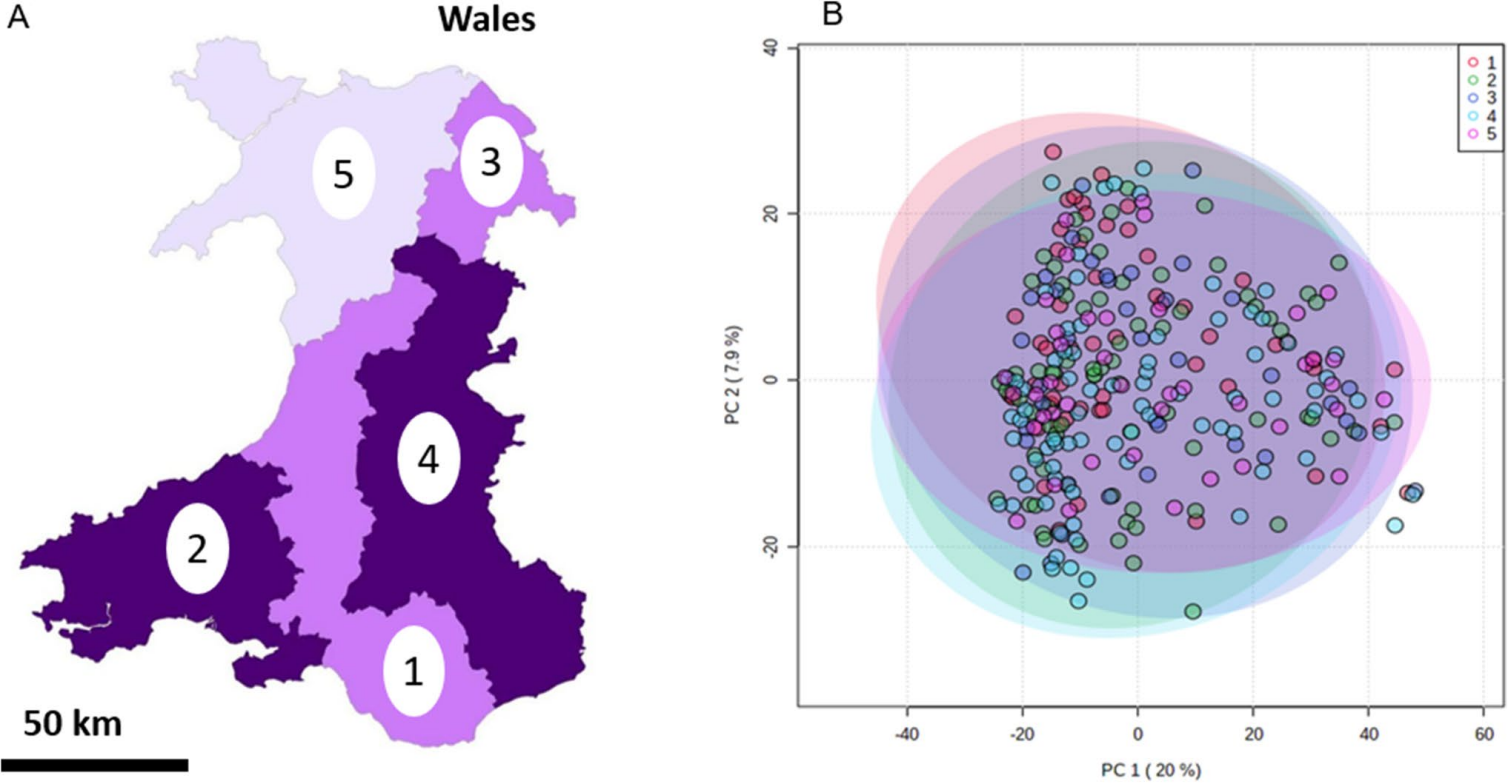
Partial least squares discriminant analysis (PLS-DA) of thoracic fluid metabolomes comparing badgers from the five Welsh risk areas. The Welsh risk areas (**A**); 1 = high risk east, 2 = high risk west, 3 = intermediate area north, 4 = intermediate area mid and 5 = low risk area. **B** PLS-DA of metabolomes related to welsh region. The shaded ellipsis represent 95% CI for each group

Finally, it was considered how geographical origins of the badgers could have influenced metabolomic variation, such as from underlying genetic differences or different diets. Badgers carcass locations were related to one of the five TB risk areas of Wales (Fig. 6A). Broadly, regions 1–4 are lower lying agricultural areas whilst region 5 is primarily a mountainous area. However, it was not possible to discriminate the samples based on the region where the samples were found (Fig. 6B).

## Discussion

The results above show little variation across the metabolomes of almost 300 wild badgers associated with anything other than sex. Region, presence of bites or causes of lymphadenopathies (other than bTB) detected at post-mortem all had little effect on the total variance of metabolites picked up by FIE-MS. It’s possible that if the region from where badgers were collected from was known in better detail then there would be a greater chance of this affecting their metabolome statistically but for this study no greater detail than the 5 TB risk areas for Wales was known (Fig. 6A). Age did have a small effect, but the most significant factor by far, as shown by the two-way ANOVA, is sex.

The mummichog algorithm looks at all the *m/z* values identified in a dataset and attempts to link *m/z*’s that appear many times in specific pathways. Then, after identifying pathways that are likely to be active in that data, it assigns tentative identifications to each *m/z* value. From the heatmap in Fig. 3 various prostanoids (thromboxanes, arachidonic acid, prostaglandins) can be seen to be significantly higher in female badgers than male ones. Various studies have shown similar sex-linked variations in prostanoids, but the association is not always clear. For example Decsi and Kennedy (2011) found arachidonic acid (and docosahexaenoic acid) similarly was lower in men than in women, while Gecse et al. (1979) found various prostaglandins (PGE2, PGF2α and PGD2) to be significantly higher in male animals. Part of the variability in results may be associated with the involvement of various prostaglandins in the oestrus cycle, with PGF2α providing a luteolytic effect in many species (Pate, 2018). The reproductive endocrinology in the badger has not been extensively studied but it is known that they can undergo embryonic diapause (Paulson & Comizzoli, 2021), leading to varying levels of progesterone through the year in females (Bonnin et al., 1978). It has also been shown that androgens exert inhibitory effects on the formation of leukotrienes in innate immune cells of humans resulting in lower leukotriene formation in male cells (Pergola et al., 2008, 2011).

## Conclusion

The major source of variation in the badger’s metabolome was linked to sex and to a much lesser extent, age. Within the female population, variation was also seen between lactating and non-lactating individuals. No significant variation was linked to the presence of bite wounds, lymph node lesions or geographical region of origin. Thus, any future metabolomic work that makes comparisons between bTB infected and non-infected badger samples using this sampling strategy need only to be sex-matched; such as comparing only males to remove any sex or lactation bias. A large scale analysis of blood samples from badgers with bTB using metabolomics would require a containment level 3 laboratory but could provide potentially invaluable insights into the pathogenesis of disease in this species and could also help identify novel biomarkers for the disease.

## Supplementary Information

Below is the link to the electronic supplementary material.

Supplementary file1 (TIF 200 KB)—The highly variable nature of the thoracic fluid samples collected

Supplementary file2 (TIF 83 KB)—Venn Diagram comparing showing the significant metabolites associated with either sex and age and their interaction as indicated by two-way ANOVA

Supplementary file3 (CSV 24 KB)

## Data Availability

The raw data are available on Mendeley Data (https://doi.org/10.17632/8m5n462gcv.1).

## Declarations

### Research involving animal participants

All samples used in this study were collected from badgers that were found dead. The carcasses were undergoing a post-mortem as part of an ongoing Welsh government study (All Wales Badger Found Dead study) to monitor levels of tuberculosis in the population, when an additional sample was taken for this study.
