# Supplementary figures and images for: Metabotyping the Welsh population of badgers based on thoracic fluid
Source: Metabolomics. 2022 May 7;18(5):30. doi: 10.1007/s11306-022-01888-6 (PMC9079023; doi:10.1007/s11306-022-01888-6)

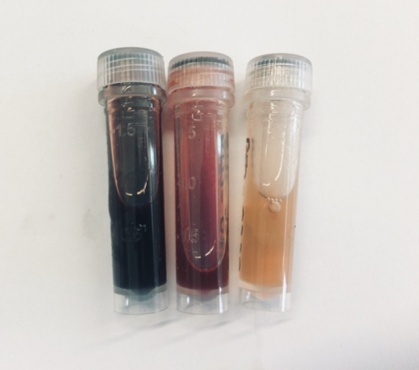

Supplement: Supplementary file 1 — Supplementary file1 (TIF 200 KB)—The highly variable nature of the thoracic fluid samples collected [file 11306_2022_1888_MOESM1_ESM.tif]

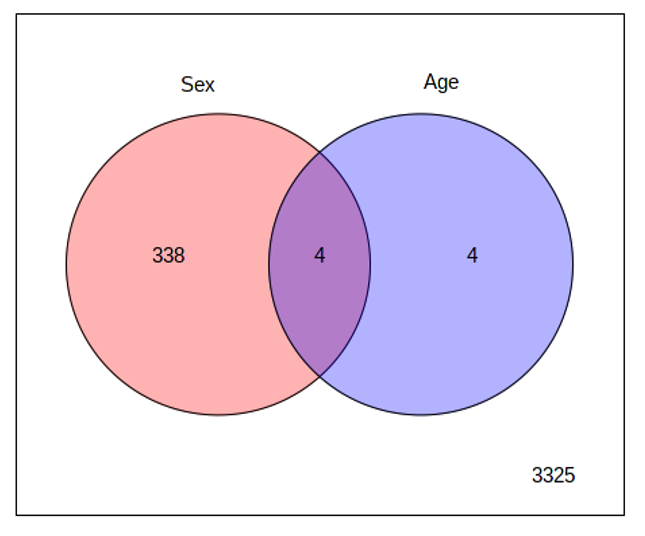

Supplement: Supplementary file 2 — Supplementary file2 (TIF 83 KB)—Venn Diagram comparing showing the significant metabolites associated with either sex and age and their interaction as indicated by two-way ANOVA [file 11306_2022_1888_MOESM2_ESM.tif]
